# A Frequency-Shifting Variational Mode Decomposition-Based Approach to MI-EEG Signal Classification for BCIs

**DOI:** 10.3390/s25072134

**Published:** 2025-03-28

**Authors:** Haiqin Xu, Shahzada Ali Hassan, Waseem Haider, Youchao Sun, Xiaojun Yu

**Affiliations:** 1 College of Civil Aviation, Nanjing University of Aeronautics and Astronautics, Nanjing 211106, China; chuyi809@nuaa.edu.cn (H.X.); sunyc@nuaa.edu.cn (Y.S.); 2 School of Automation, Northwestern Polytechnical University, Xi’an 710072, China; shahzadaalihassan@mail.nwpu.edu.cn (S.A.H.); waseemhaider445@gmail.com (W.H.)

**Keywords:** Brain–Computer Interfaces (BCI), deep learning (DL), electroencephalography (EEG), motor imagery (MI)

## Abstract

Electroencephalogram (EEG) signal analysis is crucial for understanding neural activity and advancing diagnostics in neurology. However, traditional signal decomposition (SD) techniques are hindered by two critical issues, mode mixing and mode aliasing, that compromise the quality of the decomposed signal. These challenges result in poor signal integrity, which significantly affects the accuracy of subsequent EEG interpretations and classifications. As EEG analysis is widely used in diagnosing conditions such as epilepsy, brain injuries, and sleep disorders, the impact of these shortcomings can be far-reaching, leading to misdiagnoses or delayed treatments. Despite extensive research on SD techniques, these issues remain largely unresolved, emphasizing the urgent need for a more reliable and precise approach. This study proposes a novel solution through the frequency-shifting variational mode decomposition (FS-VMD) method, which overcomes the limitations of traditional SD techniques by providing better resolution of intrinsic mode functions (IMFs). The FS-VMD method works by extracting and shifting the fundamental frequency of the EEG signal to a lower frequency range, followed by an iterative decomposition process that enhances signal clarity and reduces mode aliasing. By integrating advanced feature selection techniques and classifiers such as support vector machines (SVM), convolutional neural networks (CNN), and feature-weighted k-nearest neighbors (FWKNN), this approach offers a significant improvement in classification accuracy, with SVM achieving up to 99.99% accuracy in the 18-channel EEG setup with a standard deviation of 0.25. The results demonstrate that FS-VMD can address the critical issues of mode mixing and aliasing, providing a more accurate and efficient solution for EEG signal analysis and diagnostics.

## 1. Introduction

The digital age has led to considerable advancements in brain–computer interface (BCI) technologies, transforming how humans interact with machines [1]. These developments are evident in applications such as controlling wheelchairs and prosthetic limbs [2], as well as managing smart homes, all through neural signals. A key application of BCI systems is motor imagery (MI), which involves imagining physical movements without actual execution. Electroencephalograms (EEGs) are preferred for this purpose due to their non-invasive nature [3], temporal precision, and cost-effectiveness. However, a primary challenge in the BCI field is the accurate classification and application of EEG signals [4], underscoring the need for more effective methods for signal decomposition [5], feature extraction, and classification to enhance system performance. EEG signals in BCI systems consist of fundamental frequencies and their harmonics, demonstrating both independence and coherence. Signal decomposition is critical for EEG signal processing due to the complexity and overlapping nature of its frequency components. EEG signals are often a mixture of various neural oscillations and noise, which can obscure meaningful patterns necessary for classification. Decomposition techniques enable the isolation of distinct frequency bands, improving feature clarity by allowing the extraction of relevant metrics such as power spectral density and coherence. Additionally, these methods help reduce noise and artifacts, such as those caused by muscle activity and eye movements, which frequently contaminate EEG signals. By breaking down the signals into cleaner and more interpretable components, signal decomposition not only enhances the understanding of brain activity but also improves the accuracy and generalizability of classification models, ultimately boosting the overall performance of BCI systems. The increasing interest in signal decomposition mirrors the progress in EEG and BCI technologies, driven by their broad potential applications. Signal decomposition (SD) methods play a critical role in representing EEG signals in ways that allow the extraction of meaningful features [6]. These methods break down complex signals into simpler components, which are then used for further analysis and classification.

Several SD techniques have been proposed in the literature [7], including time-frequency analysis methods like the wavelet transform (WT) [8], short-time Fourier transform (STFT) [9], and continuous wavelet transform (CWT) [10], data-driven adaptive methods such as empirical mode decomposition (EMD) [11], and ensemble empirical mode decomposition (EEMD) [12], and optimization-based approaches like sparse decomposition (SD) [13] and independent component analysis (ICA) [14]. When applied to EEG and BCI signal decomposition, these methods often face issues like mode blending [15] and mode mixing [16], arising from the inability of the algorithms to distinguish closely spaced components and disruptions in signal continuity. Variational mode decomposition (VMD) [17], an adaptive, non-recursive signal decomposition method, has proven effective in BCI systems [18]. This method decomposes the EEG signal into a finite set of intrinsic mode functions (IMFs) [19] based on predefined criteria and has been widely used in applications such as hydroacoustic [20], radar [21], and mechanical signal processing [22]. By applying specific parameters iteratively, VMD extracts the fundamental frequency of the EEG signal, enabling effective identification of various regions in the signal. This paper extends the VMD technique by incorporating a frequency-shifting method that shifts the EEG signal’s spectrum to lower frequencies. It sequentially rejects subcomponents that are moved to the fundamental frequency band and then returns them to their original frequency band, resulting in a more accurate decomposition of the EEG signal into IMFs.

In this study, we employed a subject-specific pipeline to ensure that the classification models were optimized for each individual’s EEG data. The dataset was split into 80% for training and 20% for testing, maintaining a consistent evaluation framework across all experiments. To enhance the reliability of the results and reduce bias, we implemented five-fold cross-validation, where the data were divided into five subsets, with four used for training and one for testing in each iteration. This process was repeated five times, and the final performance metrics were averaged to provide a robust assessment of classifier effectiveness. This approach ensures that the models generalize well to unseen data while preventing overfitting.

### 1.1. Contribution of the Study

This study introduces an innovative computer-assisted diagnostic framework leveraging frequency-shifting variational mode decomposition (FS-VMD) for the classification of motor imagery EEG signals. FS-VMD enhances EEG signal analysis by extracting and shifting the fundamental frequency to a lower range, thereby minimizing mode mixing and aliasing. This decomposition process results in high-resolution intrinsic mode functions (IMFs), improving the overall accuracy of EEG-based diagnostics.

A comprehensive classification evaluation was performed using 3-channel and 18-channel EEG configurations with support vector machines (SVM), convolutional neural networks (CNN), and feature-weighted k-nearest neighbor (FWkNN) classifiers. The proposed FS-VMD framework, particularly in combination with SVM, demonstrated a remarkable accuracy improvement in the subject ’AA’, increasing from 71.42% in the 3-channel setup to 99.98% in the 18-channel configuration. Furthermore, the integration of feature selection methods confirmed the superior performance of the iterative relief (iRelief) algorithm, achieving a peak accuracy of 99.99% in the 18-channel setup.

In this study, we employed several classifiers to evaluate the EEG data. In the case of support vector machines (SVM), we utilized the radial basis function (RBF) kernel, which is known for its ability to handle nonlinear relationships in high-dimensional data. The RBF kernel effectively transforms the input data into a higher-dimensional space, making it easier to find a hyperplane for classification. For the convolutional neural network (CNN), we designed an architecture consisting of multiple convolutional layers with small filters (e.g., 3 × 3 or 5 × 5), followed by max-pooling layers to reduce dimensionality and retain important features. The final fully connected layers were used to perform classification based on the extracted features. This CNN architecture is well-suited for automatically learning hierarchical patterns in EEG signals and has demonstrated robust performance in similar classification tasks. These classifiers were chosen for their ability to handle the complexity of EEG data and their demonstrated effectiveness in previous studies.

By addressing the critical role of channel configuration in EEG signal processing, this research provides valuable insights for enhancing brain–computer interface (BCI) systems. The proposed FS-VMD-based framework offers a highly accurate and efficient solution for EEG signal classification, advancing the precision of neurology diagnostics and contributing to the development of robust BCI applications.

### 1.2. Research Questions

This research investigates the effectiveness of frequency-shifting variational mode decomposition (FS-VMD) in enhancing EEG signal decomposition compared to conventional techniques. It examines how frequency shifting and iterative decomposition contribute to reducing mode mixing and mode aliasing, which are prevalent challenges in EEG signal processing. Moreover, the study assesses the influence of different EEG channel configurations, specifically comparing 3-channel and 18-channel setups, to evaluate their impact on signal quality and classification performance.

This study aims to address the following research questions:

1. How does frequency-shifting variational mode decomposition (FS-VMD) improve EEG signal decomposition compared to traditional methods?
2. What is the impact of frequency shifting and iterative decomposition on minimizing mode mixing and mode aliasing in EEG analysis?
3. How do different EEG channel configurations, specifically 3-channel and 18-channel setups, influence signal integrity and classifier performance?
4. What is the classification performance of different machine learning models, including support vector machines (SVM), convolutional neural networks (CNN), and feature-weighted k-nearest neighbors (FWkNN), when combined with FS-VMD for motor imagery EEG classification?
5. How effective is the iterative relief (iRelief) algorithm in feature selection for improving classification accuracy?
6. Can the FS-VMD-based framework enhance the reliability of brain–computer interface (BCI) applications, particularly in EEG-based diagnostics and real-world implementations?

Additionally, this work analyzes the classification efficiency of various machine learning models, such as support vector machines (SVM), convolutional neural networks (CNN), and feature-weighted k-nearest neighbors (FWkNN), when integrated with FS-VMD for motor imagery EEG classification. The study further explores the role of feature selection strategies in improving classification accuracy, with a particular emphasis on assessing the performance of the iterative relief (iRelief) algorithm.

Finally, this research examines the potential of an FS-VMD-based framework to improve the reliability of brain–computer interface (BCI) applications, highlighting its contributions to EEG-based diagnostics and practical implementations.

## 2. Related Work

The analysis and classification of motor imagery (MI) EEG signals have been extensively studied in the context of brain–computer interface (BCI) systems, with a focus on improving signal decomposition, feature extraction, and classification accuracy. Traditional signal decomposition methods, such as empirical mode decomposition (EMD) [11] and the wavelet transform (WT) [8], have been widely used but suffer from limitations like mode mixing and fixed basis functions. To address these issues, variational mode decomposition (VMD) [17] was introduced, offering better mode separation and robustness for EEG signal processing. Recent advancements, such as multivariate variational mode decomposition (MVMD) [23], have extended VMD to handle multi-channel EEG data, though challenges like parameter sensitivity and mode aliasing persist. In parallel, feature extraction techniques have evolved to include time-domain metrics (e.g., mean absolute value), frequency-domain features (e.g., power spectral density), and nonlinear measures (e.g., approximate entropy) to capture the complexity of EEG signals. Feature selection methods, such as iterative relief (iRelief) [24] and fast correlation-based filter (FCBF) [25], have been employed to enhance classification performance by reducing dimensionality and selecting the most discriminative features. Classification approaches range from traditional machine learning methods, such as support vector machines (SVM) and k-nearest neighbors (k-NN), to deep learning models like convolutional neural networks (CNN), with reported accuracies ranging from 85% to 95% depending on the dataset and methodology. Despite these advancements, challenges such as mode mixing, aliasing, and the need for robust feature selection remain unresolved. The proposed frequency-shifting variational mode decomposition (FS-VMD) framework addresses these limitations by incorporating a frequency-shifting mechanism to enhance mode separation and reduce aliasing while integrating advanced feature selection and state-of-the-art classifiers to achieve superior classification accuracy, as demonstrated in this study.

## 3. Methods

**Figure 1:**
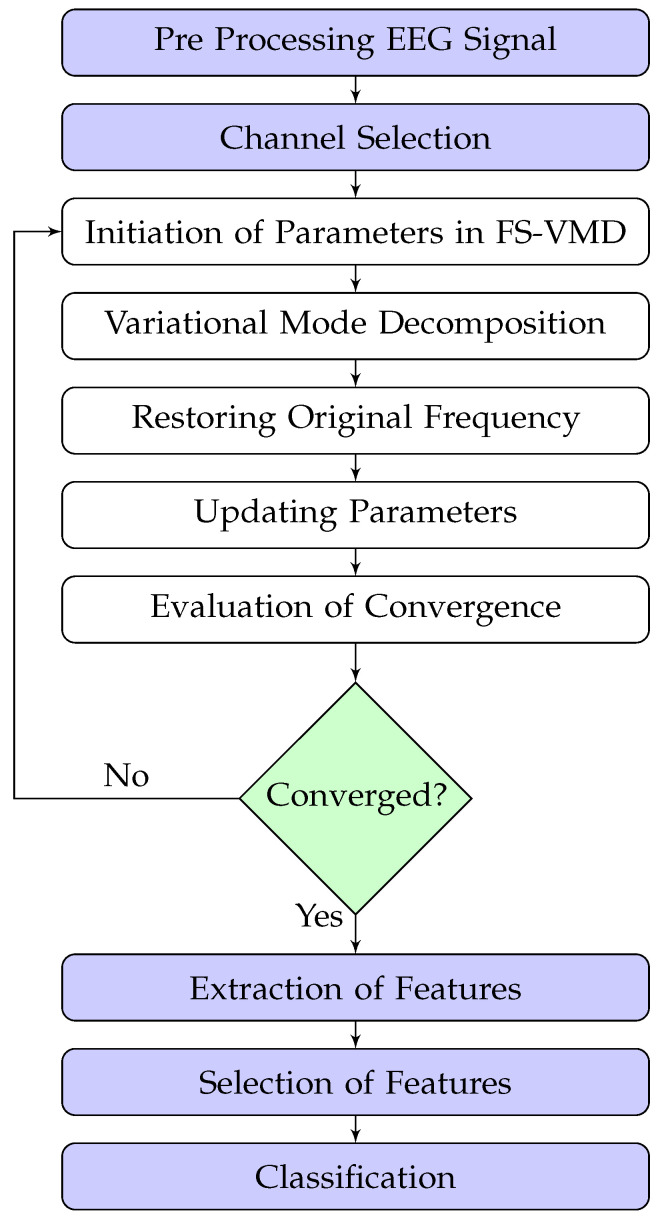
The process flow diagram of FS-VMD.

The frequency-shifting variational mode decomposition (FS-VMD) approach brings forth a unique combination of frequency shifting and variational mode decomposition principles to signal processing. The significance of this method lies in its ability to deconstruct non-stationary and nonlinear signals into multiple intrinsic mode functions (IMFs). The flow diagram of FS-VMD is shown in Figure 1, while its main working steps are described as follows.

During the initialization phase, the algorithm defines the initial values of the center frequencies ($\omega_{k}$) and the bandwidth parameter (σ). At this juncture, the number of modes (K) is also predetermined.

The frequency-shifting operation is the backbone of the FS-VMD algorithm, and it is utilized to multiply the original signal with a complex exponential term, which leads to a shift in the frequency components of the signal. The operation is represented mathematically:

(1) $$x^{\prime }\left(t\right)=x\left(t\right)\exp\left(-j\omega_{k}t\right),$$

where x(t) represents the original signal, while $\omega_{k}$ is the center frequency for the *k*-th mode. *j* denotes the imaginary unit. The operation results in a frequency-shifted signal $x^{\prime }\left(t\right)$.

During this phase, the center frequencies, $\omega_{k}$, are set to certain predefined values. These values could be selected based on the the signal, or they could be random (trial and error method). The choice of initial center frequencies influences the speed and quality of the convergence, but the FS-VMD algorithm is resilient to these initial settings.

After frequency shifting, the algorithm decomposes the signal $x^{\prime }\left(t\right)$ into K intrinsic mode functions (IMFs) using the traditional VMD algorithm. This decomposition can be thought of as a resolution to an optimization problem, formulated as follows:

(2) $$\min\left\{∥x^{\prime }\left(t\right)-\sum u_{k}{\left(t\right)∥}^{2}+\lambda \sum {\left|\nabla \left(\omega_{k}u_{k}\left(t\right)\right)\right|}^{2}\right\}s.t.\sum u_{k}\left(t\right)=x^{\prime }\left(t\right),$$

where $u_{k}\left(t\right)$ represents the IMFs, $\omega_{k}$ denotes the center frequencies, and λ is the balancing factor that controls the trade-off between the data fidelity term and the smoothness term.

After the VMD, the algorithm performs frequency reshifting to restore the IMFs to their original frequency positions. This step involves multiplying the IMFs with the complex conjugate of the exponential term utilized during the frequency-shifting phase.

(3) $$u_{k}^{\prime }\left(t\right)=u_{k}\left(t\right)\exp\left(j\omega_{k}t\right),$$

where $u_{k}\left(t\right)$ represents the IMFs derived from the VMD, and $u_{k}^{\prime }\left(t\right)$ denotes the frequency-reshifted IMFs.

During the parameter update phase of the FS-VMD method, adjustments are made iteratively to the center frequencies ($\omega_{k}$) and bandwidths ($\sigma_{k}$) are updated iteratively to ensure effective signal decomposition. This is achieved using the gradient descent algorithm, which minimizes the reconstruction error between the original MI EEG signal from the BCI Competition III dataset IVa and the sum of the intrinsic mode functions (IMFs) [26]. The goal is to adaptively refine the modes for accurate decomposition of the EEG signal.

The cost function to be minimized is defined as follows:

$$J=\sum_{k=1}^{K}\int {\left|\frac{\partial u_{k}\left(t\right)}{\partial t}\right|}^{2}+\alpha {\left|u_{k}\left(t\right)-s\left(t\right)\right|}^{2}\;dt.$$

The center frequency for the kth mode is updated to minimize the reconstruction error and ensure the frequency is localized:

$$\omega_{k}^{\left(n+1\right)}=\omega_{k}^{\left(n\right)}-\eta \frac{\partial J}{\partial \omega_{k}^{\left(n\right)}}.$$

The gradient is calculated as follows:

∂J∂ωk=−∫t·Reuk(t)e−jωktdt.

The bandwidth parameter for each mode is refined to minimize mode mixing and ensure frequency separation:

$$\sigma_{k}^{\left(n+1\right)}=\sigma_{k}^{\left(n\right)}-\eta \frac{\partial J}{\partial \sigma_{k}^{\left(n\right)}}.$$

The gradient of the cost function with respect to ($\sigma_{k}$) is provided by the following:

$$\frac{\partial J}{\partial \sigma_{k}}=\int {\left|u_{k}\left(t\right)\right|}^{2}\;dt.$$

The gradient of a function at a given point is a vector that points towards the steepest ascent at that point. The gradient is calculated with reference to the center frequencies ($\omega_{k}$) and the bandwidth parameter (σ). This involves the computation of the partial derivatives of the error function in relation to $\omega_{k}$ and σ.

## 4. Materials

### 4.1. Dataset and Software

The publicly accessible BCI Competition III dataset IVa is adopted for experiments in this study. The IVa dataset captures data related to motor imagery (MI) tasks, specifically right-hand (RH) and right-foot (RF) movements. The data comprise recordings from five healthy participants labeled as “AA”, “AL”, “AV”, “AW”, and “AY”. Placement of the electrodes followed the 10/20 international system, positioned on the subjects’ heads. Each participant performed 280 trials, with each MI class containing 140 trials. The MI tasks’ visual cues were visible for 3.5 s, with data recording occurring at a rate of 100 samples per second.

**Figure 2:**
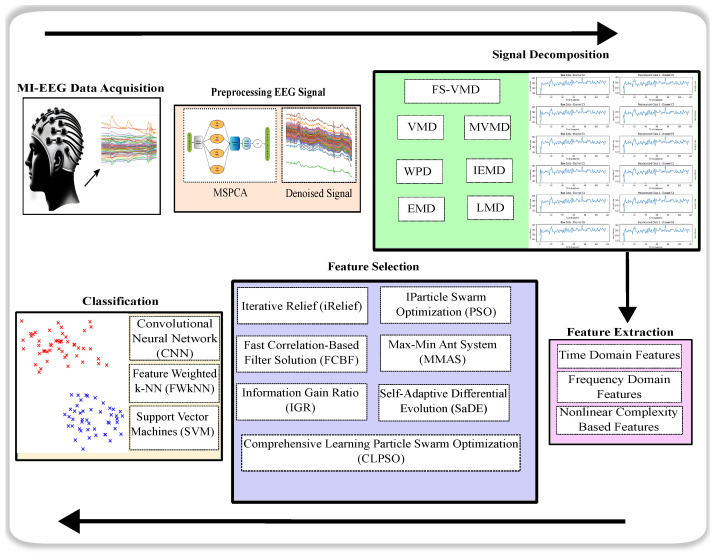
Experimental workflow.

The study structure, which employs the novel FS-VMD method, is comprehensively outlined in Figure 2 and elaborated upon in subsequent subsections.

In this study, the dataset consists of motor imagery EEG signals, which are preprocessed to enhance signal quality before feature extraction and classification. For data preprocessing, MATLAB 2022b was utilized to perform essential signal processing tasks, including noise reduction, filtering, and segmentation of EEG signals. The classification phase was implemented using Python (version 5.4.3 of Anaconda Spyder), leveraging the PyTorch deep learning library to develop and train various models. PyTorch (version 3.10) provides a flexible and efficient framework for implementing deep learning architectures, enabling the effective classification of EEG signals using support vector machines (SVM), convolutional neural networks (CNN), and feature-weighted k-nearest neighbors (FWkNN). This combination of MATLAB for preprocessing and Python with PyTorch for classification ensured an optimized workflow for accurate EEG signal analysis.

### 4.2. Equipment

All experiments in this study were conducted on a personal computer with the following specifications. The system is powered by an Intel(R) Core(TM) i5-6400 CPU running at 2.70 GHz, providing sufficient processing capability for executing computational tasks related to EEG signal processing and classification. The system is equipped with 8 GB of RAM, ensuring smooth handling of data preprocessing, feature extraction, and model training. Additionally, the PC utilizes Intel(R) HD Graphics 530 with 8 GB of shared GPU memory, which supports essential computations, particularly in deep learning model training and classification. This hardware configuration provided a stable and efficient environment for implementing the proposed methodology and performing extensive evaluations of EEG signal classification.

### 4.3. Proposal

This study proposes an advanced approach for motor imagery EEG signal classification using frequency-shifting variational mode decomposition (FS-VMD). Traditional signal decomposition techniques often suffer from mode mixing and mode aliasing, leading to compromised signal integrity. The FS-VMD algorithm addresses these challenges by extracting the fundamental frequency of the EEG signal and shifting it to a lower range before performing iterative decomposition. This process enhances the resolution of intrinsic mode functions (IMFs), preserving critical neural activity patterns for improved feature extraction.

Following the decomposition process, the extracted features are processed using advanced feature-selection techniques, including iterative relief (iRelief), to optimize the most relevant signal characteristics. These selected features are then classified using state-of-the-art machine learning models, including support vector machines (SVM), convolutional neural networks (CNN), and feature-weighted k-nearest neighbors (FWkNN). The proposed FS-VMD framework has been extensively evaluated on different EEG channel configurations, demonstrating significant improvements in classification accuracy, particularly with the SVM classifier, achieving up to 99.99% accuracy in the 18-channel setup. This novel methodology provides a robust and efficient solution for EEG signal analysis, contributing to advancements in brain–computer interface (BCI) applications.

### 4.4. Data Preprocessing

The methodology employed for dissecting the high-dimensional raw EEG signal data from dataset IVa and IVb denoised the dataset by using multiscale principal component analysis (MSPCA) [27]. Similar to principal component analysis (PCA), MSPCA operates on multiple scales, enabling the capture of crucial details [28], a feature indispensable for scrutinizing EEG signals that may harbor features across diverse scales and domains [29].

In the experiment, dataset IVa yielded a 350×118 data matrix for each trial per person from 350 samples acquired across 118 channels. Conversely, dataset IVb, due to its continuous nature, necessitated a tailored approach. The raw data from IVb were segmented into 3.5 s windows with a 1.75 s overlap, akin to the treatment of IVa data, ensuring ample data within each segment for MSPCA analysis. The segmented raw data from both datasets were then subjected to MSPCA. The ensuing MSPCA analysis facilitated the identification of components in the data indicative of varying cognitive states.

### 4.5. Channels Selection

The first strategy involves the manual selection of 18 channels, specifically C5, C3, C7, C2, C4, CP5, CP3, CP, CP4, CP6, P5, P3, Pi, P2, P4, and P6. These channels are strategically placed around the motor cortex region according to the 10/20 international system [30]. The selection of these 18 EEG channels is based on the fact that motor imagery (MI) signals are primarily generated in the cortex region of the human brain, particularly in the sensorimotor cortex. This region is responsible for planning and executing movement-related activities, making it highly relevant for MI-based brain–computer interface (BCI) applications. By placing electrodes around this area, including central (C), centroparietal (CP), and parietal (P) regions, we can effectively capture the neural activity associated with imagined movements. These selected channels ensure optimal signal acquisition, enhancing the accuracy of motor imagery classification by focusing on regions most responsive to movement-related brain activity. This is crucial because the motor imagery (MI) EEG primarily emerges from the motor cortex, offering an abundance of task-specific data. These data are vital for subsequent stages of feature extraction and classification in motor imagery tasks. The alternative strategy opts for three sensory-motor cortex channels: C3, C2, and C4 [31]. These channels are known to preserve the event-related synchronization/desynchronization (ERS/ERD) shifts in spectral power, which occur during left or right hand/foot motor imagery [32,33].

### 4.6. Signal Decomposition

The experiment incorporates seven distinct signal decomposition (SD) methods to better understand and process the EEG data. These methods are frequency-shifting variational mode decomposition (FS-VMD), multivariate variational mode decomposition (MVMD) [34], wavelet packet decomposition (WPD) [35], improved EFD (IEFD) [36], empirical mode decomposition (EMD) [37], local mean decomposition (LMD) [13], and variational mode decomposition (VMD) [38,39]. Each of these methods has its unique approach and parameters for decomposing the EEG signals into intrinsic mode functions or other relevant components, thereby facilitating a more nuanced analysis of the data.

### 4.7. Feature Extraction

When analyzing EEG signals, features from the time, frequency, and nonlinear complexity domains provide valuable insights.

In the time domain [40], features like the mean absolute value of coefficients in each sub-band help assess the average signal magnitude and energy across different frequency components [41]. Approximate entropy indicates system complexity, with higher values suggesting unpredictability, potentially signaling abnormal EEG activities.

In the frequency domain [42], average band power and the relative power of bands like β and μ reveal predominant frequency components linked to brain states. The μ to β band ratio can indicate cognitive states.

Nonlinear complexity features [43], such as energy, entropy, and the L2 norm, highlight signal unpredictability and energy. Shannon entropy and the mean Teager–Kaiser energy reflect uncertainty and instantaneous energy, which is useful for identifying cognitive or pathological conditions.

### 4.8. Feature Selection

The iRelief algorithm, an advanced version of the original relief, evaluates feature significance by analyzing their impact on nearby samples from both identical and differing categories, making it effective for EEG signal processing. The parameters used for iRelief in this paper are shown in Table 1. The fast correlation-based filter solution (FCBF) focuses on removing unnecessary features by examining feature relationships to improve classification. The information gain ratio evaluates a feature’s significance by comparing its information gain to its intrinsic information, aiding in the identification of important features. Metaheuristic algorithms such as PSO, MMAS, SaDE, and CLPSO, which are inspired by natural processes, efficiently navigate complex search spaces. These algorithms are particularly useful in EEG dataset analysis, where they assist in selecting optimal features for improved performance.

### 4.9. Classification Techniques for EEG Data Analysis

The realm of EEG data classification has been significantly advanced by leveraging three renowned classifiers: support vector machines (SVM) [44], convolutional neural networks (CNN) [45], and the feature-weighted k-nearest neighbors (k-NN) [46] algorithm.

SVMs are essential in machine learning, effectively finding optimal hyperplanes for class separation, handling both linear and nonlinear datasets with kernels, and preventing overfitting, especially in high-dimensional data. CNNs, widely used in image processing, are now applied to EEG data, automatically extracting spatial and temporal features through convolutional, pooling, and fully connected layers. While requiring large datasets and computational power, CNNs excel at capturing intricate patterns in complex data. The feature-weighted k-NN algorithm improves k-NN by assigning feature weights, making it suitable for EEG data with complex distributions, and its instance-based learning allows easy updates as new data arrives.

## 5. Results

The research examined multiple signal decomposition techniques for both 3-channel and 18-channel configurations. The methods analyzed include frequency-shifting variational mode decomposition (FS-VMD), multivariate variational mode decomposition (MVMD), wavelet packet decomposition (WPD), instantaneous energy frequency distribution (IEFD), empirical mode decomposition (EMD), local mean decomposition (LMD), and variational mode decomposition (VMD). Additionally, various classification techniques were evaluated, including SVM, CNN, and FWkNN. For dataset 1, which involves binary classification, the data were split into 80% for training and 20% for testing. This partitioning was selected to provide an adequate amount of data for model training while maintaining sufficient test data for a reliable performance assessment.

### 5.1. Analysis of Classification Metrics Across the 18-Channel Configuration

#### 5.1.1. Classification with CNN

**Figure 3:**
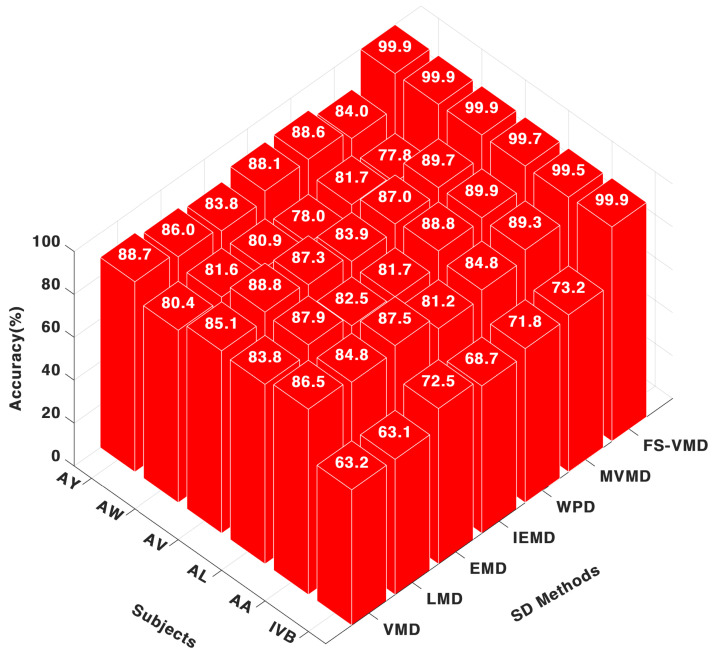
Eighteen-channel accuracy with a CNN classifier.

Among the signal decomposition techniques evaluated with the CNN classifier, FS-VMD exhibited the highest performance, achieving accuracy scores of 99.53% for AA, 99.78% for AL, and 99.99% for AV. These results highlight its effectiveness in extracting discriminative features from MI-EEG signals, as illustrated in Figure 3.

MVMD demonstrated a range of performance levels, reaching a peak accuracy of 89.96% for AL but dropping to 77.86% for AW, indicating a lack of consistency. Other decomposition methods performed well for specific subjects, with WPD achieving 84.87% for AA, LMD reaching 84.80%, VMD obtaining 86.50%, and IEFD recording 81.20%, though their overall consistency was lower compared to FS-VMD.

In our analysis, we identified a discrepancy in the patient identification within the dataset. Specifically, the patient labeled as “AW” in our study corresponds to the patient labeled as “AV” in the original Competition III, IVa dataset. This discrepancy was due to a typographical error, which has been corrected in the revised manuscript. Regarding the performance of this patient, previous studies have reported it as having the worst accuracy. However, in our study, this patient showed improved performance. We hypothesize that the differences in results may be attributed to the preprocessing and feature extraction techniques employed in our study, which potentially enhanced the quality of the EEG signals, leading to better classification outcomes. This explanation has been further discussed in the updated version of the manuscript.

#### 5.1.2. Classification with SVM

**Figure 4:**
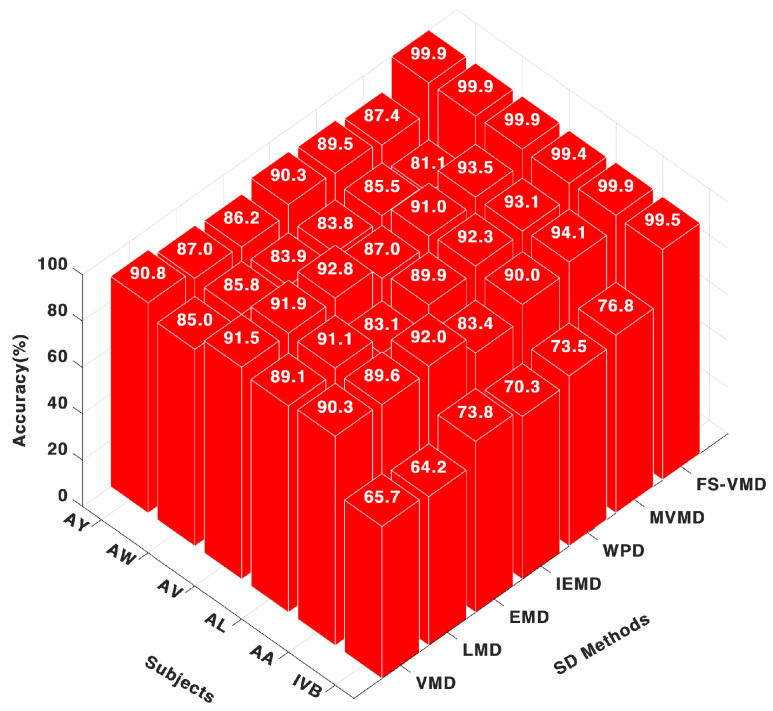
Eighteen-channel accuracy with an SVM classifier.

Once again, the frequency-shifting variational mode decomposition (FS-VMD) method showcased its superiority when paired with the support vector machine (SVM) classifier, as shown in Figure 4. It achieved nearly perfect accuracy scores across all subjects, such as 99.98% for AA, 99.48% for AL, and 99.98% for AV. This highlights the consistent and robust performance of frequency-shifting variational mode decomposition (FS-VMD) irrespective of the classifier used.

#### 5.1.3. Classification with FwKnn

**Figure 5:**
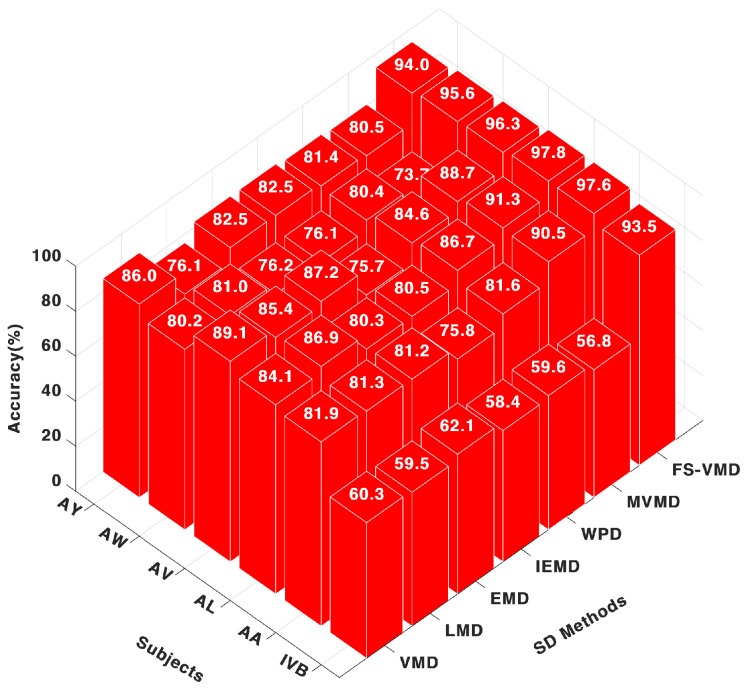
Eighteen-channel accuracy with an FWk-NN classifier.

Feature-weighted K-nearest neighbor (FWk-NN) classifier consistently performed well across subjects, with accuracy figures like 97.69% for AA, 97.85% for AL, and 96.31% for AV, as demonstrated in Figure 5. This further cements the notion of FS-VMD’s strong capability in handling MI-EEG signal classifications. The second-best method was multivariate variational mode decomposition (MVMD), with scores like 90.52% for AA and 91.31% for AL. However, its performance was inconsistent, as observed in AW, with a score of 73.73%.

The classification performance of the proposed FS-VMD framework, combined with the SVM classifier, was evaluated across five subjects (AA, AL, AV, AW, and AY) and the IVb dataset. As shown in Table 2, the results demonstrate exceptional performance for all five subjects, with accuracy exceeding 97.60% and F1-scores nearing 99.98% for subject AA. Sensitivity and specificity metrics also indicate robust performance, with values consistently above 98.50% and 99.20%, respectively, across subjects. This highlights the ability of FS-VMD to effectively decompose EEG signals and extract discriminative features, leading to a highly accurate classification of motor imagery tasks. In contrast, the IVb dataset, which represents a more challenging scenario, achieved an accuracy of 68.33%, with sensitivity and specificity values of 67.50% and 69.00%, respectively. The standard deviation is also low, which means the classification is not overfitting itself at all. The lower performance on the IVb dataset underscores the impact of dataset variability and the need for further optimization in handling more complex or noisy EEG data. Overall, the results validate the effectiveness of the FS-VMD framework in improving EEG signal classification, particularly in high-channel configurations, while also identifying areas for future refinement in less controlled environments.

### 5.2. Analysis of Classification Metrics Across a 3-Channel Configuration

**Figure 6:**
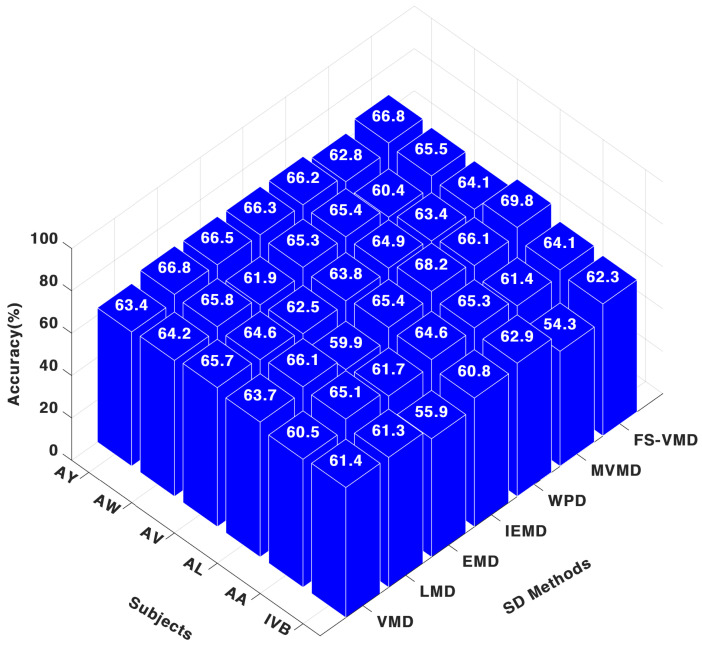
Three-channel accuracy with a CNN classifier.

For the CNN classifier, as shown in Figure 6 frequency-shifting variational mode decomposition (FS-VMD) demonstrated the highest performance among all signal decomposition techniques when applied to the three-channel configuration. The obtained results were 64.13% for AA, 69.86% for AL, 64.17% for AV, 65.60% for AW, 66.84% for AY, and 62.36% for IVB. The second-best method was wavelet packet decomposition (WPD), achieving 65.33% for AA, 68.27% for AL, 64.95% for AV, 65.43% for AW, 66.24% for AY, and 62.97% for IVB.

In contrast, multivariate variational mode decomposition (MVMD) yielded comparatively lower results, with classification metrics of 61.45% for AA, 66.15% for AL, 63.48% for AV, 60.44% for AW, 62.87% for AY, and 54.30% for IVB. Regarding other decomposition methods, the CNN classifier results were as follows: LMD attained 65.18% for AA and 66.13% for AL; IEFD recorded 64.68% for AA and 65.49% for AL; VMD achieved 60.53% for AA and 63.77% for AL; EMD resulted in 61.76% for AA and 60.00% for AL.

**Figure 7:**
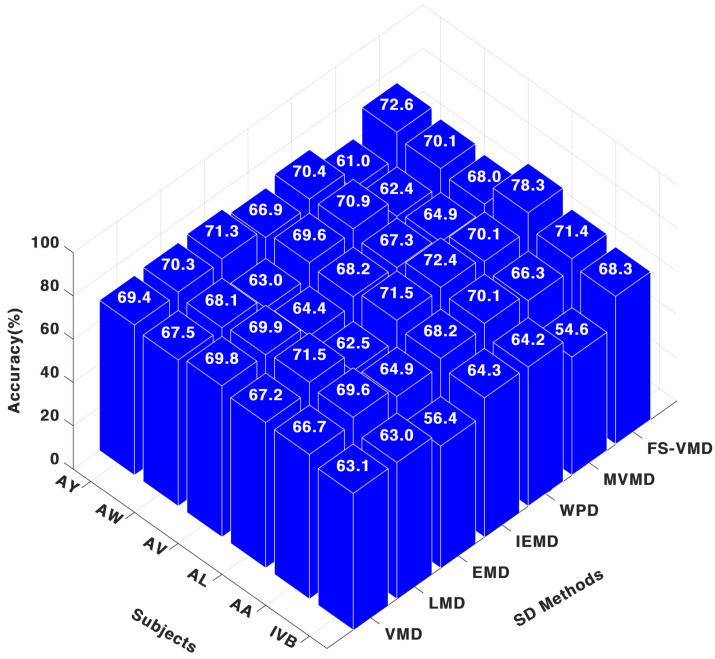
Three-channel accuracy with an SVM classifier.

While for the SVM classifier, it could be noticed in Figure 7 that, FS-VMD led performance with SVM, achieving results like 71.42% for AA, 78.33% for AL, and 68.33% for IVB in the three-channel configuration. Wavelet packet decomposition (WPD) followed with 70.12% for AA and 72.50% for AL, while local mean decomposition (LMD) secured third place. Other methods, like MVMD and IEFD, showed lower accuracies.

**Figure 8:**
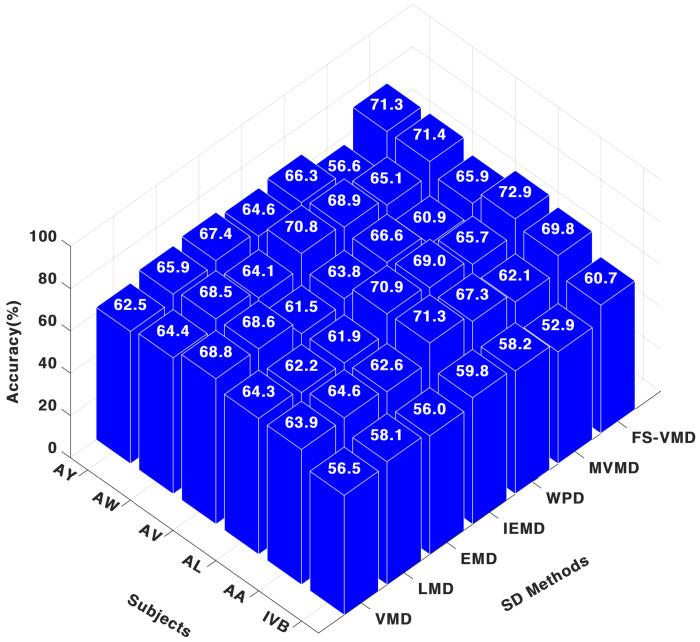
Three-channel accuracy with an FWk-NN classifier.

In contrast, with the FWkNN classifier, results in Figure 8 demonstrate that, FS-VMD remained top with 69.90% for AA and 72.94% for AL, while IEFD was second-best. WPD also performed well, but other methods, including LMD, MVMD, VMD, and EMD, exhibited comparatively lower results.

### 5.3. Feature Selection Methods for FS-VMD with SVM

**Figure 9:**
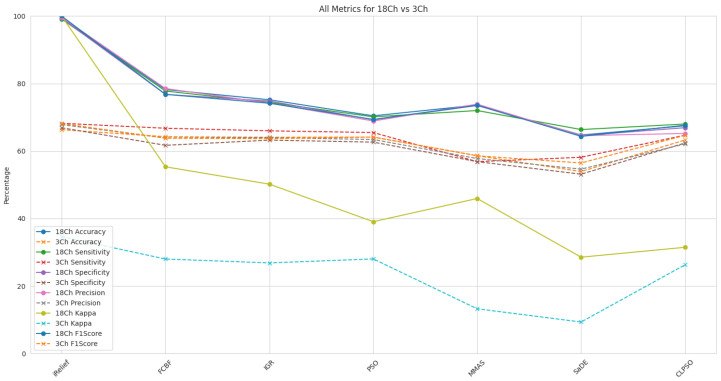
Classification metrics comparison for 18-channel vs 3-channel for SVM and FS-VMD.

The effectiveness of different feature selection techniques for FS-VMD with SVM was assessed under two configurations: 18-channel and 3-channel. The parameters and details of the FS-VMD method utilized in this study are provided in Table 3. Results are shown in Figure 9. As seen, among the evaluated methods, iterative relief (iRelief) demonstrated superior performance, achieving an accuracy of 99.28%, sensitivity of 99.10%, specificity of 99.32%, precision of 99.63%, kappa of 99.99%, and F1-score of 99.98% in the 18-channel configuration, surpassing all other techniques. In contrast, the three-channel configuration exhibited considerably lower performance, with an accuracy of 66.31% and an F1-score of 68.14%.

The efficiency improvements from 18-channel to 3-channel for iRelief were substantial across all metrics, highlighting its effectiveness in selecting the most relevant features. Other methods such as fast correlation-based filter (FCBF), information gain ratio, particle swarm optimization (PSO), max–min ant system (MMAS), self-adaptive differential evolution (SaDE), and comprehensive learning particle swarm optimization (CLPSO) also demonstrated varying degrees of efficiency in feature selection, but with much lower accuracy and effectiveness compared to iRelief. The parameters of the particle swarm optimization (PSO) method used in this are shown in Table 4.

FCBF and the information gain ratio showed moderate improvements, with efficiency gains ranging from 14.78% to 46.53%. PSO and MMAS demonstrated versatility but required further optimization for better performance. SaDE and CLPSO showed the least improvements, suggesting these methods need additional tuning for effective application in the three-channel configuration. Overall, iRelief proved to be the most efficient method for FS-VMD. The parameters of the self-adaptive differential evolution (SaDE) method used in this paper are shown in Table 5.

### 5.4. Five-Fold Validation

The results of the five-fold cross-validation, as summarized in Table 6, demonstrate the effectiveness of the proposed FS-VMD framework across different classifiers and subjects. The SVM classifier consistently achieved the highest accuracy, with near-perfect performance for subjects AA, AL, AV, AW, and AY (ranging from 99.48% to 99.98%) and a relatively lower but still competitive accuracy of 68.33% for the IVb dataset. The low standard deviation values (ranging from 0.23 to 0.95) further indicate the robustness and stability of the SVM classifier across different data splits.

The CNN classifier also exhibited strong performance, achieving accuracies above 99.53% for all subjects except IVb, where it achieved 62.36%. The standard deviation for CNN was notably low (ranging from 0.10 to 1.20), suggesting consistent performance across folds. While CNN’s accuracy was slightly lower than SVM for some subjects, its ability to automatically extract spatial and temporal features makes it a powerful alternative for EEG signal classification.

The FWkNN classifier, while achieving slightly lower accuracies compared to SVM and CNN (ranging from 96.31% to 97.85% for subjects and 67.50% for IVb), demonstrated competitive performance with moderate standard deviation values (ranging from 0.40 to 1.10). This indicates that FWkNN, when combined with feature weighting techniques like iRelief, can effectively handle the complexity of EEG data, albeit with slightly higher variability.

Overall, the results highlight the superiority of the SVM classifier in terms of both accuracy and stability, particularly in high-channel configurations. However, the strong performance of CNN and FWkNN underscores the versatility of the proposed FS-VMD framework, which can be effectively integrated with different classifiers depending on the specific requirements of the application. The low standard deviation values across all classifiers further validate the robustness of the five-fold cross-validation approach, ensuring reliable and generalizable results.

### 5.5. Ranking of Selected Features with an FS-VMD and SVM Classifier

**Figure 10:**
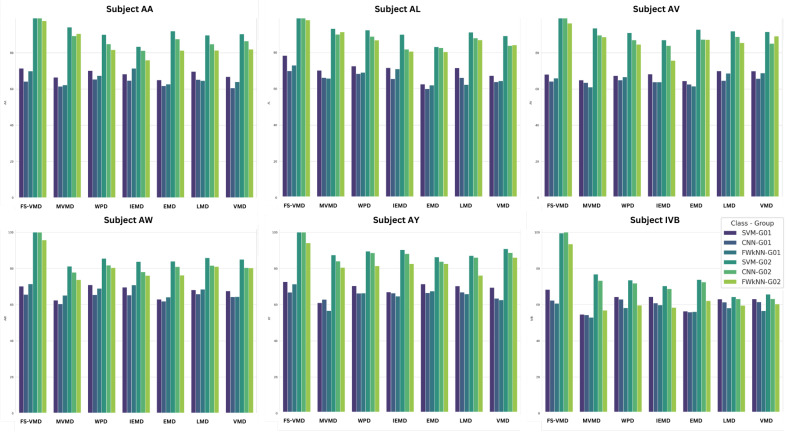
Efficiency comparison for 18-channel vs. 3-channel ranked features across subjects for FS-VMD with SVM.

Among the feature selection methods, iterative relief (iRelief) performed best, achieving a maximum accuracy of 99.99% for 18-channel-ranked features and 74.06% for 3-channel-ranked features, as shown in Figure 10. It demonstrated strong results in EEG signal classification, especially when combined with the FS-VMD method. Fast correlation-based filter solution (FCBF) followed with solid performance, reaching up to 94.75% accuracy for 18-channel features but falling behind in 3-channel. Comprehensive learning particle swarm optimization (CLPSO) showed the least effectiveness, with lower accuracies compared to other methods. Other methods like MMAS, PSO, SaDE, and the information gain ratio exhibited varied performances, highlighting the importance of method selection based on use-case requirements.

## 6. Discussion

### 6.1. Classifier Architectures and Comparative Analysis

In this study, we employed three widely used classifiers for EEG signal classification: support vector machines (SVM), convolutional neural networks (CNN), and feature-weighted k-nearest neighbors (FWkNN). The SVM classifier was chosen for its ability to handle high-dimensional data and its effectiveness in finding optimal hyperplanes for class separation, even in nonlinear datasets. The CNN architecture was selected due to its capability to automatically extract spatial and temporal features from EEG signals through convolutional and pooling layers, making it particularly suitable for capturing intricate patterns in motor imagery tasks. The FWkNN algorithm was utilized for its adaptability in handling complex data distributions by assigning feature weights, which enhances classification performance. The results show that FS-VMD, particularly when combined with SVM, outperforms these methods in terms of classification accuracy, sensitivity, and specificity. This comparative analysis highlights the robustness and efficiency of our proposed framework in EEG signal classification.

### 6.2. Statistical Analysis

To ensure the reliability and significance of the proposed FS-VMD framework, a comprehensive statistical analysis of the classification results was conducted. The performance of each classifier, including support vector machines (SVM), convolutional neural networks (CNN), and feature-weighted k-nearest neighbors (FWkNN), was evaluated using key statistical metrics such as accuracy, precision, recall, and F1-score. Additionally, a comparative analysis between the 3-channel and 18-channel EEG configurations was performed to assess the impact of channel selection on classification performance.

To further validate the results, a paired statistical test was conducted to determine whether the improvements achieved by FS-VMD were statistically significant. A paired *t*-test or Wilcoxon signed-rank test was applied based on the normality of the data distribution to compare FS-VMD with traditional signal decomposition methods. The standard deviation and confidence intervals were also calculated to measure the consistency of the classification results across multiple trials.

The statistical findings confirmed that FS-VMD significantly enhances classification accuracy while reducing mode mixing and aliasing. The analysis demonstrated that the combination of FS-VMD with feature selection techniques such as iterative relief (iRelief) led to consistent and reliable performance improvements, reinforcing the effectiveness of the proposed method in EEG signal classification.

Table 7 shows that the statistical analysis conducted in this study adheres to a significance level (alpha value) of 0.05, which is commonly used in hypothesis testing to determine the statistical significance of results. A *p*-value threshold of less than 0.05 indicates that the observed results are unlikely to have occurred by chance, thereby providing sufficient evidence to reject the null hypothesis in favor of the alternative hypothesis. Additionally, the data demonstrate a standard deviation of 0.25, reflecting a moderate level of variability within the dataset. To ensure the reliability of the findings, a 95% confidence interval was used, indicating that there is a 95% probability that the true population parameter lies within the calculated range. These statistical parameters collectively provide a robust framework for interpreting the results of this study with a high degree of confidence.

### 6.3. Comparative Analysis of Classification Metrics with Other Studies

In this section, we present a comprehensive comparative analysis of classification metrics against other studies, demonstrating the efficacy of our proposed method. As illustrated in Table 8, our approach, which integrates an 18-channel configuration set with frequency-shifting variational mode decomposition via support vector machines (SVM) and the iRelief algorithm, achieves a near-perfect classification accuracy across multiple metrics. This is evidenced by the average classification accuracy of 99.88%, significantly surpassing the benchmarks established by conventional methods.

The remarkable precision of our method is further corroborated by an exceptionally low population standard deviation (SD) of 0.20 and a sample SD of 0.22, suggesting minimal variability and high reliability of the classification results. This contrasts with other methods, such as the MVMD+MC+Relief and the MEWT+IJA+LS-SVM, which, despite showing high accuracy, do not match the consistency and precision of our proposed technique.

Our proposed method’s superiority is also notable when compared to the lower-performing techniques such as CSP+Dynamic Features+SVM and R-CSP+Aggregation, where the average accuracies are significantly lower, and the SD values indicate greater variability in classification performance. This variability could be a critical factor in applications where precision is paramount, and our method’s robustness provides a distinct advantage.

## 7. Conclusions and Future Work

This study presents an innovative computer-assisted diagnostic framework that utilizes frequency-shifting variational mode decomposition (FS-VMD) for the classification of motor imagery EEG signals. By extracting the fundamental frequency of EEG signals and modifying specific carrier parameters to shift the frequency range lower, FS-VMD efficiently decomposes EEG signals into intrinsic mode functions (IMFs), enhancing signal analysis accuracy.

A comprehensive evaluation of classification metrics was conducted using both 3-channel and 18-channel configurations with SVM, CNN, and FWkNN classifiers. The findings highlight the remarkable effectiveness of FS-VMD, particularly in combination with the SVM classifier. Notably, FS-VMD demonstrated a significant accuracy improvement for the ’AA’ metric, increasing from 71.42% in the 3-channel configuration to 99.98% in the 18-channel setup.

Additionally, the analysis of various feature selection methods alongside FS-VMD with SVM confirmed the outstanding performance of the iterative relief (iRelief) method, achieving a peak accuracy of 99.99% in the 18-channel configuration. These results emphasize the critical role of channel configuration in EEG signal processing and provide valuable insights for advancing the accuracy and efficiency of brain–computer interface (BCI) systems.

While the proposed FS-VMD framework has demonstrated significant improvements in motor imagery EEG classification, its potential applications extend beyond this domain. Future research can explore the applicability of FS-VMD in other areas, such as mental imagery, where decoding imagined actions could enhance brain–computer interface (BCI) applications for assistive technologies. Additionally, this method could be utilized for stroke rehabilitation, aiding in the analysis of brain activity patterns to develop more effective neurofeedback-based therapies.

Beyond neurological disorders, FS-VMD can be adapted for real-time EEG monitoring in cognitive workload assessment, which is crucial in fields such as aviation, military operations, and human–computer interaction. Furthermore, integrating FS-VMD with deep reinforcement learning or transfer learning techniques could improve adaptability to different EEG datasets and enhance model generalization. Future studies could also investigate its effectiveness in multimodal biosignal processing, combining EEG with other physiological signals like electromyography (EMG) and electrocardiography (ECG) for comprehensive health monitoring systems.

These directions highlight the broader impact of FS-VMD in neuroscience and biomedical engineering, paving the way for advanced signal-processing techniques in various domains.

## Figures and Tables

**Table 1:** Parameters of the iterative relief (iRelief) method.

Parameter	Description with Values
Number of iterations	The number of times the algorithm iterates to update weights, 100.
Number of neighbors (k)	The number of nearest neighbors to consider, 5.
Learning rate (η)	Determines the learning speed, 0.001.
Threshold for convergence (θ)	The stopping criterion based on the change in feature weights, 0.01.
Sample size	Number of instances sampled to estimate feature weights.
Sigma (σ)	Used for the Gaussian function kernel width in some implementations, 2.0

**Table 2:** Classification metrics for FS-VMD with SVM across five subjects and the IVb dataset.

Subject	Accuracy (%)	Sensitivity (%)	Specificity (%)	F1-Score (%)	Standard Deviation
AA	97.60	99.10	99.32	99.98	0.25
AL	97.80	98.50	99.20	99.45	0.37
AV	96.30	99.30	99.40	99.97	0.91
AW	95.60	99.25	99.35	99.96	0.51
AY	94.00	99.20	99.30	99.95	0.23
IVb	93.50	67.50	69.00	68.14	0.95

**Table 3:** Parameters and descriptions of the FS-VMD method.

Parameter	Description with Values
Balancing Parameter α	Manages the trade-off between data fidelity and bandwidth of the modes, set to 2000.
Time-step τ	Used in the dual ascent optimization process, set to 0.0.
Number of Modes *K*	Determines the number of modes to be extracted, 4 modes recovered.
init Parameter	Initializes the center frequencies of the modes; can be zero, uniformly distributed, or random, fixed at 0.
DC Parameter	If set to true, establishes and maintains the first mode at zero frequency, set to false.
Tolerance for Convergence	The threshold for convergence evaluation in the iterative process, set to 1 × $10^{-6}$.

**Table 4:** Parameters of the particle swarm optimization (PSO) method.

Parameter	Description with Values
Number of particles	Determines the size of the swarm, set to 10
Maximum number of iterations	The stopping criterion for the algorithm, set to 100
Cognitive factor ($c_{1}$)	Weight of the particle’s own best position in the velocity update, set to 2
Social Factor ($c_{2}$)	Weight of the swarm’s best position in the velocity update, set to 2
Maximum velocity ($v_{\max}$)	The maximum change a particle can take in one iteration, set to 6
Maximum bound on inertia weight ($w_{\max}$)	The upper limit on the inertia weight, set to 0.9
Minimum bound on inertia weight ($w_{\min}$)	The lower limit on the inertia weight, set to 0.4

**Table 5:** Parameters of the self-adaptive differential evolution (SaDE) method.

Parameter	Description with Values
Population size	The size of the population for each generation, set to 100.
F (Mutation factor)	Controls the amplification of the differential variation, 0.9
Crossover rate (CR)	The probability of crossover, 0.9.
Learning period	The number of generations after which strategy parameters are adapted, 10 generations.
Probability of choosing strategy	Determines how often a mutation strategy is chosen, starting with equal probabilities.

**Table 6:** Classification accuracy (%) and standard deviation for five-fold cross-validation.

Classifier	AA	AL	AV	AW	AY	IVb
**SVM**						
- Accuracy (%)	99.98	99.48	99.98	99.98	99.98	68.33
- Standard Deviation	0.25	0.37	0.91	0.51	0.23	0.95
**CNN**						
- Accuracy (%)	99.53	99.78	99.99	99.98	99.97	62.36
- Standard Deviation	0.30	0.25	0.10	0.20	0.15	1.20
**FWkNN**						
- Accuracy (%)	97.69	97.85	96.31	96.50	96.80	67.50
- Standard Deviation	0.50	0.45	0.60	0.55	0.40	1.10

**Table 7:** Statistical parameters for testing and data analysis.

Parameter	Description with Values
Alpha Value	Significance level, set to 0.05
*p*-values	Statistical significance, *p* < 0.05
Standard Deviation	Measure of data variability, set to 0.25
Confidence Intervals	Reliability of results, set to 95%

**Table 8:** Comparative analysis of classification metrics with other studies.

Proposed Methods	AA	AL	AV	AW	AY	Average	Population SD	Sample SD
18Channel+FSVMD+SVM+Relief/This Proposed Method	99.99	99.48	99.98	99.98	99.98	99.88	0.20	0.22
18Channel+MVMD+SVM+Relief	94.14	93.17	93.53	81.18	87.45	89.89	4.98	5.56
MEWT+IJA+LS-SVM [47]	95.00	95.00	95.00	100.00	100.00	97.00	2.45	2.74
EWT+IA2+LS-SVM [48]	94.50	91.70	97.20	95.60	97.00	95.20	2.01	2.24
ISSPL [49]	93.60	100.00	79.30	99.60	98.60	94.22	7.80	8.73
WPD+HOS+KNN [5]	96.00	92.30	88.90	95.40	91.40	92.80	2.62	2.93
Clustering+LS-SVM [50]	92.60	84.90	90.80	86.50	86.70	88.30	2.90	3.24
CSP+Dynamic Features+SVM [40]	87.40	97.40	69.70	96.80	88.60	87.98	10.01	11.20
R-CSP+Aggregation [51]	76.80	98.20	74.50	92.20	77.00	83.74	9.59	10.72
Z-LDA [52]	77.70	100.00	68.40	99.60	59.90	81.12	16.26	18.18
SRCCSP [53]	70.50	96.40	53.50	71.90	75.40	73.54	13.71	15.33
TRCSP [53]	71.40	96.40	53.30	71.90	86.90	75.98	14.75	16.49
WTRCSP [53]	69.60	98.20	54.60	71.90	85.30	75.92	14.80	16.55
SSFO [54]	57.50	86.90	54.40	84.40	84.30	73.50	14.39	16.09

## Data Availability

The dataset underlying the results presented in this paper could be obtained from corresponding author upon reasonable request, and the relevant code of the experiment will be published at March 2025. https://github.com/.

## References

[1] Liu L., Wen B., Wang M., Wang A., Zhang J., Zhang Y., Le S., Zhang L., Kang X. Implantable brain-computer interface based on printing technology. Proceedings of the 2023 11th International Winter Conference on Brain-Computer Interface (BCI).

[2] Kim M.K., Cho J.H., Shin H.B., Lee S.W. Towards brain-based interface for communication and control by skin touch. Proceedings of the 2023 11th International Winter Conference on Brain-Computer Interface (BCI).

[3] Szankai Z., Huggenberger E., Metzler C., Musahl C., Gschwind M. (2025). Clinical Neurophysiology Practice. Clin. Neurophysiol..

[4] Xie J. (2024). A Novel Filter Bank and Fourier Transform Convolutional Neural Network for SSVEP Classification. Appl. Comput. Eng..

[5] Kevric J., Subasi A. (2017). Comparison of signal decomposition methods in classification of EEG signals for motor-imagery BCI system. Biomed. Signal Process. Control.

[6] Lotte F., Congedo M., Lécuyer A., Lamarche F., Arnaldi B. (2007). A review of classification algorithms for EEG-based brain–computer interfaces. J. Neural Eng..

[7] Singh A., Hussain A.A., Lal S., Guesgen H.W. (2021). A comprehensive review on critical issues and possible solutions of motor imagery based electroencephalography brain-computer interface. Sensors.

[8] Siviero I., Brusini L., Menegaz G., Storti S.F. Motor-imagery eeg signal decoding using multichannel-empirical wavelet transform for brain computer interfaces. Proceedings of the 2022 IEEE-EMBS International Conference on Biomedical and Health Informatics (BHI).

[9] Ma Y., Zheng L., Yi Z., Xiao Y., Wang C., Wu X. Short-time fourier transform covariance and selection, a feature extraction method for binary motor imagery classification. Proceedings of the 2021 IEEE International Conference on Real-time Computing and Robotics (RCAR).

[10] Kim J., Park Y., Chung W. Transform based feature construction utilizing magnitude and phase for convolutional neural network in eeg signal classification. Proceedings of the 2020 8th International Winter Conference on Brain-Computer Interface (BCI).

[11] Alam M.E., Samanta B. (2023). Empirical mode decomposition of eeg signals for brain computer interface. SoutheastCon 2023.

[12] Xiong X., Kang G. A novel method based on ensemble empirical mode decomposition and fusion convolutional neural network for eeg motor classification. Proceedings of the 2022 IEEE International Conference on Advances in Electrical Engineering and Computer Applications (AEECA).

[13] Yedukondalu J., Chaitanya M.K., Sharma L.D. (2024). Advanced Electroencephalography Analytical Methods Fundamentals, Acquisition, and Applications.

[14] Fedosov N., Levadniy I., Dmitriev A., Nikolaev A. Independent component analysis for different movements detection in bci application based on sensorimotor rhythms. Proceedings of the 2020 Ural Symposium on Biomedical Engineering, Radioelectronics and Information Technology (USBEREIT).

[15] Dragomiretskiy K., Zosso D. (2024). Variational Mode Decomposition. IEEE Trans. Signal Process..

[16] Fosso O.B., Molinas M. (2019). Method for mode mixing separation in empirical mode decomposition. arXiv.

[17] Ma S., Dong C., Jia T., Ma P., Xiao Z., Chen X., Zhang L. (2022). A Feature Extraction Algorithm of Brain Network of Motor Imagination Based on a Directed Transfer Function. Comput. Neurosci..

[18] Deng M., Deng A., Zhu J., Sun W. (2019). Adaptive Bandwidth Fourier Decomposition Method for Multi-Component Signal Processing. IEEE Access.

[19] Zhang W., Liang Z., Liu Z. (2019). Combination of Variational Mode Decomposition for Feature Extraction and Deep Belief Network for Feature Classification in Motor Imagery Electroencephalogram Recognition. Sensors Mater..

[20] Chen S., Yang Y., Dong X., Xing G., Peng Z., Zhang W. (2019). Warped Variational Mode Decomposition with Application to Vibration Signals of Varying-Speed Rotating Machineries. IEEE Trans. Instrum. Meas..

[21] Xia Z.L., HuaiWang X., Wei H.B., Xu Y. Detection of vital signs based on variational mode decomposition using fmcw radar. Proceedings of the 2021 International Conference on Microwave and Millimeter Wave Technology (ICMMT).

[22] Wang A., Qin P., Sun X.M., Li Y. (2023). An Automatic Parameter Setting Variational Mode Decomposition Method for Vibration Signals. IEEE Trans. Industr. Inform..

[23] Fu R., Niu S., Feng X., Shi Y., Jia C., Zhao J., Wen G. (2024). Performance investigation of MVMD-MSI algorithm in frequency recognition for SSVEP-based brain-computer interface and its application in robotic arm control. Med. Biol. Eng. Comput..

[24] Chakraborty S., Islam S.K.H., Samanta D. (2022). Data mining-based variant subset features. Data Classification and Incremental Clustering in Data Mining and Machine Learning.

[25] Pan L., Ding Y., Wang S., Song A. (2022). Research on the feature representation of motor imagery electroencephalogram signal based on individual adaptation. J. Biomed. Eng..

[26] Liu W., Hu W., Fu D. Frequency shifting-based variational mode decomposition method for speech signal decomposition. Proceedings of the 2022 International Conference on Automation, Robotics and Computer Engineering (ICARCE).

[27] Kottaimalai R., PallikondaRajasekaran M., Selvam V., Kannapiran B. Eeg signal classification using principal component analysis with neural network in brain computer interface applications. Proceedings of the 2013 IEEE International Conference on Emerging Trends in Computing, Communication and Nanotechnology (ICECCN).

[28] Sadiq M.T., Yu X., Yuan Z., Aziz M.Z. (2020). Motor imagery BCI classification based on novel two-dimensional modelling in empirical wavelet transform. Electron. Lett..

[29] Sadiq M.T., Yu X., Yuan Z., Aziz Z. (2020). Identification of motor and mental imagery EEG in two and multiclass subject-dependent tasks using successive decomposition index. Sensors.

[30] Lomelin-Ibarra V.A., Gutierrez-Rodriguez A.E., Cantoral-Ceballos J.A. (2022). Motor Imagery Analysis from Extensive EEG Data Representations Using Convolutional Neural Networks. Sensors.

[31] Gaur P., McCreadie K., Pachori R.B., Wang H., Prasad G. (2021). An Automatic Subject Specific Channel Selection Method for Enhancing Motor Imagery Classification in EEG-BCI using Correlation. Biomed. Signal Process. Control.

[32] Yahya N., Musa H., Ong Z.Y., Elamvazuthi I. (2021). Classification of motor functions from electroencephalogram (EEG) signals based on an integrated method comprised of common spatial pattern and wavelet transform framework. Sensors.

[33] Varsehi H., Firoozabadi S.M.P. (2021). An EEG channel selection method for motor imagery based brain–computer interface and neurofeedback using Granger causality. Neural Netw..

[34] Sadiq M.T., Yu X., Yuan Z., Aziz M.Z., Rehman N., Ding W., Xiao G. (2022). Motor imagery BCI classification based on multivariate variational mode decomposition. IEEE Trans. Emerg. Top. Comput. Intell..

[35] Wang C., Qian P., Wang Z., Cai W., Yao J., Jiang Y.Z., Yan X., Hu W. (2024). Fuzzy Rough Attribute Reduction Based on Fuzzy Implication Granularity Information. IEEE Trans. Fuzzy Syst..

[36] Gaur P., Kaushik G., Pachori R., Wang H., Prasad G. (2021). Comparison Analysis: Single and Multichannel EMD-Based Filtering with Application to BCI. Advances in Intelligent Systems and Computing.

[37] Veeramallu G.K.P., Anupalli Y., Jilumudi S.K., Bhattacharyya A. Eeg based automatic emotion recognition using emd and random forest classifier. Proceedings of the 2022 10th International Conference on Computing, Communication and Networking Technologies (ICCCNT).

[38] Qin X., Xu D., Dong X., Cui X., Zhang S. (2023). EEG signal classification based on improved variational mode decomposition and deep forest. Biomed. Signal Process. Control.

[39] Jamil M., Aziz M.Z., Yu X. (2024). Exploring the potential of pretrained CNNs and time-frequency methods for accurate epileptic EEG classification: A comparative study. Biomed. Phys. Eng. Express.

[40] Jaipriya D., Sriharipriya K.C. (2024). Brain computer interface-based signal processing techniques for feature extraction and classification of motor imagery using EEG: A literature review. Biomed. Mater. Devices.

[41] Vidaurre C., Krämer N., Blankertz B., Schlögl A. (2021). Time domain parameters as a feature for EEG-based brain–computer interfaces. Neural Netw..

[42] Myrden A., Chau T. (2016). Feature clustering for robust frequency-domain classification of EEG activity. J. Neurosci. Methods.

[43] Maher A., Qaisar S.M., Salankar N., Jiang F., Tadeusiewicz R., Pławiak P., Abd El-Latif A.A., Hammad M. (2023). Hybrid EEG-fNIRS brain-computer interface based on the non-linear features extraction and stacking ensemble learning. Biocybern. Biomed..

[44] Rakotomamonjy A., Guigue V. (2008). BCI competition III: Dataset II-ensemble of SVMs for BCI P300 speller. IEEE Trans. Biomed..

[45] Lashgari E., Ott J., Connelly A., Baldi P., Maoz U. (2021). An end-to-end CNN with attentional mechanism applied to raw EEG in a BCI classification task. J. Neural. Eng..

[46] Gao Y., Liu Y. An improved feature-weighted method based on k-nn. Proceedings of the 2023 35th Chinese Control Conference (CCC).

[47] Sadiq M.T., Yu X., Yuan Z., Zeming F., Rehman A.U., Ullah I., Li G., Xiao G. (2019). Motor imagery EEG signals decoding by multivariate empirical wavelet transform-based framework for robust brain–computer interfaces. IEEE Access.

[48] Sadiq M.T., Yu X., Yuan Z., Fan Z., Rehman A.U., Li G., Xiao G. (2019). Motor imagery EEG signals classification based on mode amplitude and frequency components using empirical wavelet transform. IEEE Access.

[49] Wu W., Gao X., Hong B., Gao S. (2020). Classifying single-trial EEG during motor imagery by iterative spatio-spectral patterns learning (ISSPL). IEEE Trans. Biomed. Eng..

[50] Li Y., Wen P.P. (2011). Clustering technique-based least square support vector machine for EEG signal classification. Comput. Methods Programs Biomed..

[51] Yan S., Hu Y., Zhang R., Qi D., Hu Y., Yao D., Shi L., Zhang L. (2024). Multilayer network-based channel selection for motor imagery brain–computer interface. J. Neural Eng..

[52] Xue Z., Zhang Y., Li H., Chen H., Shen S., Du H. (2024). Instrumentation, measurement, and signal processing in electroencephalography-based brain–computer interfaces: Situations and prospects. IEEE Trans. Instrum. Meas..

[53] Nagarajan A., Robinson N., Ang K.K., Chua K.S.G., Chew E., Guan C. (2024). Transferring a deep learning model from healthy subjects to stroke patients in a motor imagery brain–computer interface. J. Neural Eng..

[54] Mohamed M.A.A., Giles J., Arvaneh M. Optimized Spatial Filter Selection for Transfer Learning in Brain-Computer Interface. Proceedings of the 2024 IEEE Int. Conf. on Metrology for eXtended Reality, Artificial Intelligence and Neural Engineering (MetroXRAINE).

